# Leukemia inhibitory factor enhances the development and subsequent blastocysts quality of yak oocytes *in vitro*

**DOI:** 10.3389/fvets.2022.997709

**Published:** 2022-09-21

**Authors:** Tian Zhao, Yangyang Pan, Qin Li, Tianyi Ding, Robert Niayale, Tongxiang Zhang, Jinglei Wang, Yaying Wang, Ling Zhao, Xiaohong Han, Abdul Rasheed Baloch, Yan Cui, Sijiu Yu

**Affiliations:** ^1^College of Veterinary Medicine, Gansu Agricultural University, Lanzhou, China; ^2^Gansu Province Livestock Embryo Engineering Research Center, Lanzhou, China; ^3^School of Veterinary Medicine, University for Development Studies, Tamale, Ghana; ^4^Panjwani Center for Molecular Medicine and Drug Research, International Center for Chemical and Biological Sciences, University of Karachi, Karachi, Pakistan

**Keywords:** leukemia inhibitory factor, oocyte, mitochondria, reactive oxygen species, actin, apoptosis, blastocyst quality

## Abstract

Leukemia inhibitory factor (LIF) is a multipotent cytokine of the IL-6 family which plays a critical role in the maturation and development of oocytes. This study evaluated the influence of LIF on the maturation and development ability of yak oocytes, and the quality of subsequent blastocysts under *in vitro* culture settings. Different concentrations of LIF (0, 25, 50, and 100 ng/mL) were added during the *in vitro* culture of oocytes to detect the maturation rate of oocytes, levels of mitochondria, reactive oxygen species (ROS), actin, and apoptosis in oocytes, mRNA transcription levels of apoptosis and antioxidant-related genes in oocytes, and total cell number and apoptosis levels in subsequent blastocysts. The findings revealed that 50 ng/mL LIF could significantly increase the maturation rate (*p* < 0.01), levels of mitochondria (*p* < 0.01) and actin (*p* < 0.01), and mRNA transcription levels of anti-apoptotic and antioxidant-related genes in yak oocytes. Also, 50 ng/mL LIF could significantly lower the generation of ROS (*p* < 0.01) and apoptosis levels of oocytes (*p* < 0.01). In addition, blastocysts formed from 50 ng/mL LIF-treated oocytes showed significantly larger total cell numbers (*p* < 0.01) and lower apoptosis rates (*p* < 0.01) than the control group. In conclusion, the addition of LIF during the *in vitro* maturation of yak oocytes improved the quality and the competence of maturation and development in oocytes, as well as the quality of subsequent blastocysts. The result of this study provided some insights into the role and function of LIF *in vitro* yak oocytes maturation, as well as provided fundamental knowledge for assisted reproductive technologies in the yak.

## Introduction

The yak (*Bos grunniens*) is an ancient species of the Tibetan plateau that lives year-round at high altitudes (1) and provides important subsistence and economic resources such as meat, milk, hide, hair, and dung for Tibetans and other nomadic people (2). Yaks are seasonal estrus animals, usually coming into estrus between July and October (3, 4). Because of their low reproductive performance (one litter in 2 years or 2 L in 3 years) (1), yak populations are gradually decreasing year by year. This affects the living standards of Tibetan people and is not conducive to species diversity in China. The employment of assisted reproductive techniques to enhance the yak population is an excellent alternative. The *in vitro* maturation of cultured yak oocytes and the *in vitro* development of early embryos are essential to assisted reproductive technologies, but numerous obstacles hinder their growth. Therefore, it is highly vital to discover the appropriate mechanism of *in vitro* maturation of yak oocytes and the *in vitro* development of early embryos.

Oocyte quality is a vital element for effective oocyte fertilization and preimplantation embryo development. Following ovulation, whether *in vivo* or *in vitro*, oocytes produce reactive oxygen species (ROS) due to a lack of adenosine triphosphate (ATP), which down-regulates anti-apoptotic proteins such as Bcl-2 and induces apoptosis (5, 6). There are various crucial actors such as mitochondria, ROS, and actin that affect oocyte maturation and development. Mitochondria are organelles in mammalian cells that play a crucial role in oocyte growth and function, and their activity may be utilized as a key signal to identify oocyte quality (7). Mitochondria play a key role in cellular metabolism and epigenetics (8, 9), where they interact with the nucleus to impact oocyte maturation and preimplantation embryonic development (10, 11). Furthermore, mitochondrial malfunction may raise the quantity of ROS, which can play a significant role in the apoptotic process by controlling apoptotic signaling factors such as ROS (5, 12). ROS are a class of short-lived, highly reactive small molecules (13). They are produced as a byproduct of aerobic metabolism and contain hydrogen peroxide (H_2_O_2_), superoxide anion (O${}_{2}^{-}$), and hydroxyl radicals (OH·) (14). The intracellular ROS content is at a stable level through regulation. It has been shown that low to moderate doses of ROS are considered essential in the regulation of normal physiological functions involved in cell cycle processes such as proliferation, differentiation, migration and cell death (15). Excessive ROS are produced in the cell when the antioxidant detoxification system fails to regulate intracellular ROS levels at a low level, causing damage to proteins, nucleic acids, lipids, cell membranes, and organelles (e.g., mitochondria) (13, 16), exacerbating oocyte apoptosis (17), and reducing oocyte fertility (18). Actin is one of the numerous and highly conserved protein groups found in most eukaryotic cells which plays an important role in many cellular functions (19). Actin alters various states involved in important processes such as nuclear localization, germinal vesicle transit and rupture, spindle migration and anchoring, spindle rotation, chromosomal segregation, and polar body extrusion during meiosis in mammalian oocytes (20, 21).

Leukemia inhibitory factor (LIF) is a potent cytokine that belongs to the IL-6 family of cytokines (22). LIF is involved in several crucial phases of mammalian reproduction. LIF has been found to have a key function in mammalian follicle growth, ovulation, oocyte meiosis, maturation, and embryo development and implantation (23–26). However, there are few studies on the ability of LIF to influence oocyte development and maturation through the modulation of oocyte mitochondria, ROS, and actin levels. The study sought to determine the regulatory effects of LIF on mitochondria, ROS, actin, and apoptosis in yak oocytes, which consequently alters the developmental ability of yak oocytes and the quality of subsequent blastocysts.

## Materials and methods

### Chemicals and reagents

Unless otherwise stated, all chemicals and reagents were purchased from Sigma Aldrich (St. Louis, MO, USA).

### Acquisition and selection of yak oocytes

Ovaries were collected from a commercial slaughterhouse (Xining, Qinghai, China) and stored at 30°C in sterile saline (0.9% NaCl) with two antibiotics (100 IU/mL penicillin and 0.05 mg/mL streptomycin), and sent to the laboratory within 4 h (4, 27). Upon arrival at the laboratory, ovarian samples were cleaned three times with sterile saline containing two antibiotics at 35°C to minimize external contamination. Follicular fluid was collected by extracting follicles at a diameter of 3–8 mm from the surface of the ovary using an 18G needle. The cumulus-oocyte complexes (COCs) were washed with an oocyte picker under a microscope to eliminate leftover contaminants. At the same time, unexpanded cumulus-oocyte complexes with homogenous cytoplasm surrounded by three or more layers of cumulus cells were chosen for the subsequent analysis.

### *In vitro* maturation culture and LIF treatment of yak cumulus-oocyte complexes

The immature cumulus-oocyte complexes were put in maturation media and cultivated in a 38°C, 5% CO_2_ humidified incubator for 24 h. The maturation fluid consisted of TCM-199 (Gibco, Grand Island, NY, USA), 10% FBS, 10 μg/mL follicle-stimulating hormone (FSH), 10 μg/mL Luteinizing hormone (LH) and 1 μg/mL β-Estradiol (E_2_) (28). The final concentrations of LIF (LIF1050, Sigma Aldrich, USA) were 0 ng/mL, 25 ng/mL, 50 ng/mL and 100 ng/mL by adding leukemia inhibitory factor to the maturation medium. The maturation solution was applied to a four-well plate, 400 μL per well, and incubated in a CO_2_ incubator.

### Parthenogenetic activation of yak oocytes and *in vitro* culture of embryos

After 24 h of culture, the cumulus cells enclosing the oocytes were digested with 0.1% hyaluronidase to get bare oocytes. The oocyte samples were put in 5 μM ionomycin and incubated in the incubator for 5 min. After washing, they were put in 2 mM 6-dimethylaminopurine and incubated in the incubator for 4 h (29, 30). The activated oocytes were washed three times before being transferred to synthetic oviduct fluid and cultured in a humidified incubator at 38.5°C and 5% CO_2_. After 8 d, blastocysts of each experimental group were taken for further apoptosis detection assays to count the total cell number of blastocysts.

### Detection of mitochondrial distribution in yak oocytes

Oocytes mitochondrial distribution was detected using MitoTracker^®^ Mitochondrion-Selective Probes (M22426, Invitrogen, CA). According to the manufacturer's instructions, the probe was adjusted to the working solution concentration of 200 nM and then incubated. The oocyte samples were transferred to the working solution and incubated at 37°C for 30 min in the dark. After washing 3 times, it was transferred to a glass slide and images were taken using a fluorescence inverted microscope.

### Detection of ROS in yak oocytes

Oocytes ROS levels were measured using a reactive oxygen species assay kit (S0033, Beyotime, China). The trial procedure was carried out exactly in line with the manufacturer's instructions. Briefly, the cleaned oocytes were incubated in DCFA-DA (1:5,000) in the dark at 37°C for 20 min. After washing 3 times, it was transferred to a glass slide and images were taken using a fluorescence inverted microscope.

### Detection of actin in yak oocytes

Actin was detected using the Phalloidin-iFluor^™^ 594 Conjugate Kit (23122, AAT Bioquest). According to the manufacturer's instructions, the oocyte samples were immersed in 4% paraformaldehyde for 30 min and then rinsed 3 times for 5 min each. The working solution was formed into microdroplets, and the oocytes were transferred to the microdroplets and incubated at room temperature for 45 min in the dark. After washing three times, the oocytes were placed on a glass slide, covered with a coverslip, and photographed using a fluorescent inverted microscope.

### Apoptosis detection of yak oocytes

The early apoptosis level of oocytes was measured using the Annexin V-FITC apoptosis kit (C1062L, Beyotime, China). According to the instructions, the Annexin V-FITC working solution was prepared by mixing 2.5 μL Annexin V-FITC with 97.5 μL Annexin V-FITC binding solution. The oocyte samples were transferred to the Annexin V-FITC working solution and incubated at room temperature for 20 min in the dark. The oocytes were then put on glass plates and images were taken using a fluorescent inverted microscope.

### Detection of binding ability of yak oocyte and sperm

Yak sperm was purchased from the Centre of Livestock Reproductive and Developmental in Qinghai province of China, which was frozen in 0.25-ml straws. The method was optimized according to a procedure previously reported (31, 32). Briefly, yak frozen sperm from a single straw were extracted, thawed in 37°C water for 1 min, cleaned 3 times in DPBS, and centrifuged at 1,000 g for 5 min. The sperm at the bottom of the centrifuge tube were transferred to the capacitation solution prepared in advance, kept at the bottom of the centrifuge tube, and the sperm capacitated for 1 h using the swim-up approach. The sperm density was increased to 1 × 10^6^/mL, and 400 μL of each well was inserted into a four-well plate. Mature COCs were put in a four-well plate, 40 per well, and incubated in a 38°C, 5% CO_2_ incubator for 1.5 h. After the sperm and eggs had combined, they were preserved in 4% paraformaldehyde for 30 min. The bound sperm surrounding the oocytes were stained with DAPI and quantified by using a fluorescent inverted microscope.

### Detection apoptosis level and total cell number of blastocysts

The apoptosis level of blastocysts before implantation was detected using the *In Situ* Cell Death Detection Kit (11684795910, Roche). According to the manufacturer's instructions, briefly, the cultivated blastocysts were washed, deposited in 4% paraformaldehyde for 30 min, and permeabilized in permeabilization solution for 30 min. The embryos were transferred to a TUNEL working solution (Enzyme Solution: Label Solution = 1:9 mix) and kept in a 37°C, 5% CO_2_ humidified incubator for 1 h in the dark. DAPI was used to stain the nuclei and incubated at room temperature for 5 min in the dark. The blastocysts were placed on glass slides, covered with coverslips, observed under a fluorescence microscope, and photographed. The total number of cells and the number of apoptotic cells in the samples were counted and used to calculate the apoptotic rate.

### Fluorescence microscopy imaging and fluorescence intensity analysis

Samples for immunofluorescence staining experiments were imaged using an Olympus IX81 microscope. The excitation wavelength of the fluorescence excitation block contains three sections: the ultraviolet region (340–380 nm), the blue light region (460–500 nm), and the green light region (500–570 nm). The configuration settings of the inverted microscope and the fluorescent excitation block under the same detection were consistent. Quantitative analysis was done by evaluating the fluorescence intensity of the samples using Image J (NIH, Bethesda, MD, US).

### RNA extraction and quantitative real-time PCR

Total RNA was extracted using the Micro Elute Total RNA Kit (Omega, US). cDNA was obtained *via* reverse transcription using the GoScript^™^ Reverse Transcription Kit (Promega, US). The operational procedures are carried out in full conformity with the manufacturer's instructions. Quantitative real-time PCR (qRT-PCR) were conducted in 96-well plates using TB Green^®^ Premix Ex Taq™ II (TaKaRa, Tokyo, Japan) and the LightCycler^®^ 96 System (Roche, Basel, Switzerland). Reaction volume: 2 μL of cDNA, 0.8 μL (10 μM) of upstream and downstream primers, 10 μL of the enzyme, and 6.4 μL of water to make a 20 μL system. Reaction conditions: pre-deformation at 95°C for 30 s, denaturation at 95°C for 5 s, annealing at 60°C for 34 s, extension at 72°C for 30 s, 45 cycles, and chilling at 4°C. Genes mRNA levels were normalized to glyceraldehyde 3-phosphate dehydrogenase (GAPDH), and the relative mRNA expression was calculated using the 2^−ΔΔCt^ method. All primer sequences are listed in Table 1.

**Table 1:** Gene-special primers for qRT-PCR.

**Genes**	**Forward primer sequence (5^′^–3^′^)**	**Reverse primer sequence (5^′^–3^′^)**	**Gene ID**
*Bax*	GCTGTGGACACAGACTCTCC	CCCCAGTTGAAGTTGCCGTC	102279740
*Bcl-2*	TGAGTTCGGAGGGGTCATGT	AGGTGCCGGTTCAGGTACTC	102268592
*Caspase-3*	TACTTTTCCTGGCGAAATGC	TTGCATGAAAAGCAGAATCG	408016
*Survivin*	CCTGGCAGCTCTACCTCAAG	TAGGGTCGTCATCTGGTTCC	414925
*SOD2*	TTGCTGGAAGCCATCAAACGT	AATCTGTAAGCGTCCCTGCTC	281496
*Gpx4*	TGTGCTCGCTCCATGCACGA	CCTGGCTCCTGCCTCCCAA	286809
*GAPDH*	GGGTCATCATCTCTGCACCT	TGGTCATAAGTCCCTCCACG	102275759

### Statistical analysis

Data were analyzed using SPSS 26.0 (Statistical Product and Service Solutions, US) using one-way ANOVA. Each experiment was performed at least 3 independent times. Data are presented as mean ± standard error of the mean (Mean ± SEM). Unless otherwise stated, ^*^p < 0.05 and ^**^p < 0.01.

## Results

### Effects of LIF on maturation of yak oocytes

**Figure 1:**
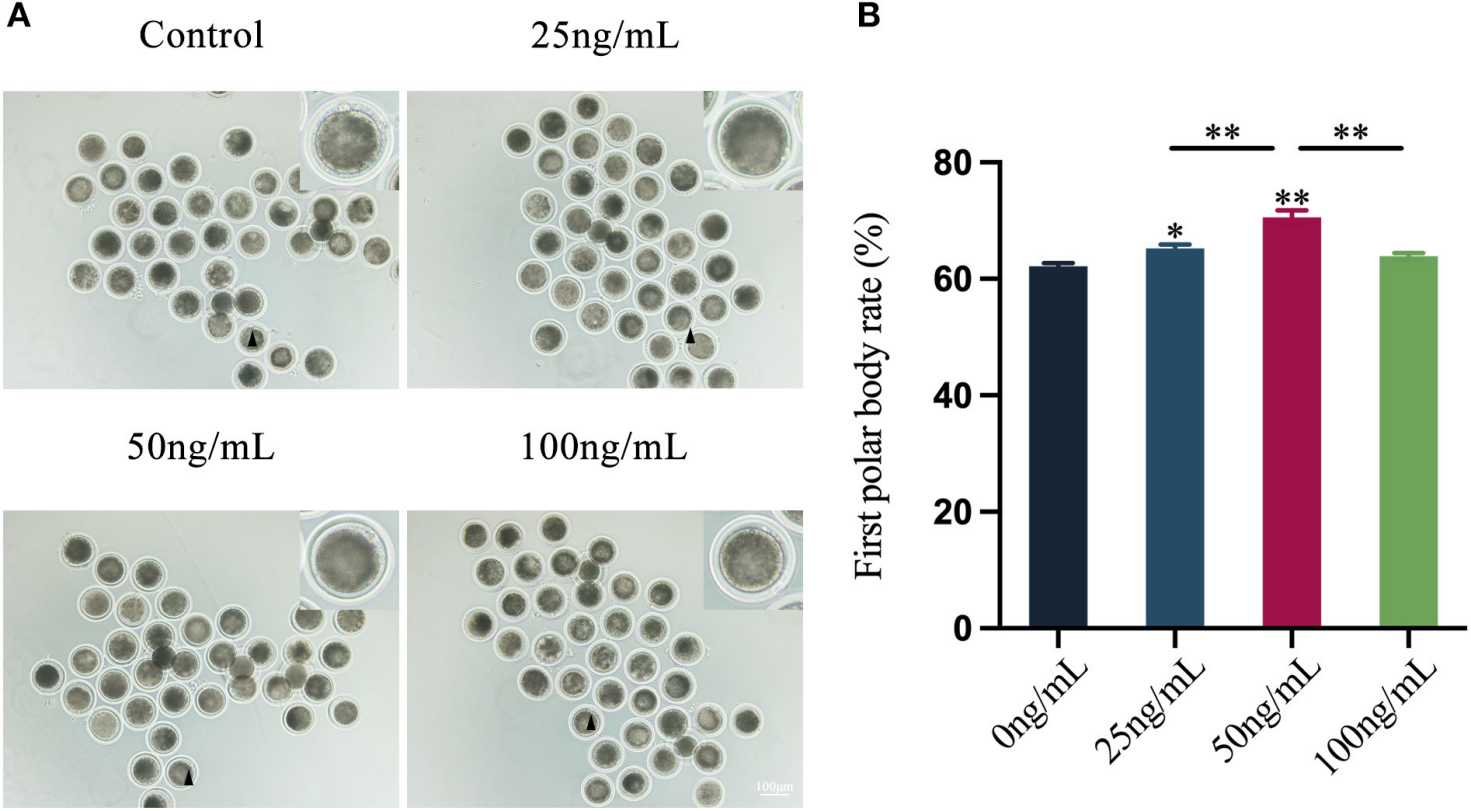
Effects of different concentrations of LIF (0, 25, 50, and 100 ng/mL) on yak oocytes *in vitro* maturation. **(A)** Yak oocytes growth status. Yak cumulus oocyte complexes were matured and cultured for 24 h *in vitro*, and the cumulus cells were digested to reveal the growth state of naked oocytes. Bar = 100 μm. **(B)** Statistics of yak oocyte maturation rate. The first polar body burst rate of oocytes in each experimental group was recorded and counted under the microscope, and the data was expressed as Mean ± SEM. * or ** on the bar graph indicates a significant difference (*p < 0.05 and **p < 0.01).

After the immature COCs were cultured in the maturation solution for 24 h, the mature status of COCs was evaluated under the microscope. The COCs cultured for 24 h were digested with hyaluronidase, and after gently pipetting, naked oocytes were obtained. The exposure of the first polar body could be observed under a microscope (Figure 1A), which signifies that the oocytes had matured. The results showed that adding LIF to the maturation fluid could enhance the oocytes maturation rate (Figure 1B). The first polar body rate of oocytes in the 50 ng/mL LIF-treated group was significantly higher than that in the other three groups (control group, 25 ng/mL LIF-treated group, and 100 ng/mL LIF-treated group, *p* < 0.01), meanwhile which in the 25 ng/mL LIF-treated group was significantly higher than that in the control group (*p* < 0.05). The first polar body rate of oocytes in the 100 ng/mL LIF-treated group was not significantly different from that in the control group (*p* > 0.05).

### Effects of LIF on mitochondrial levels of yak oocytes

**Figure 2:**
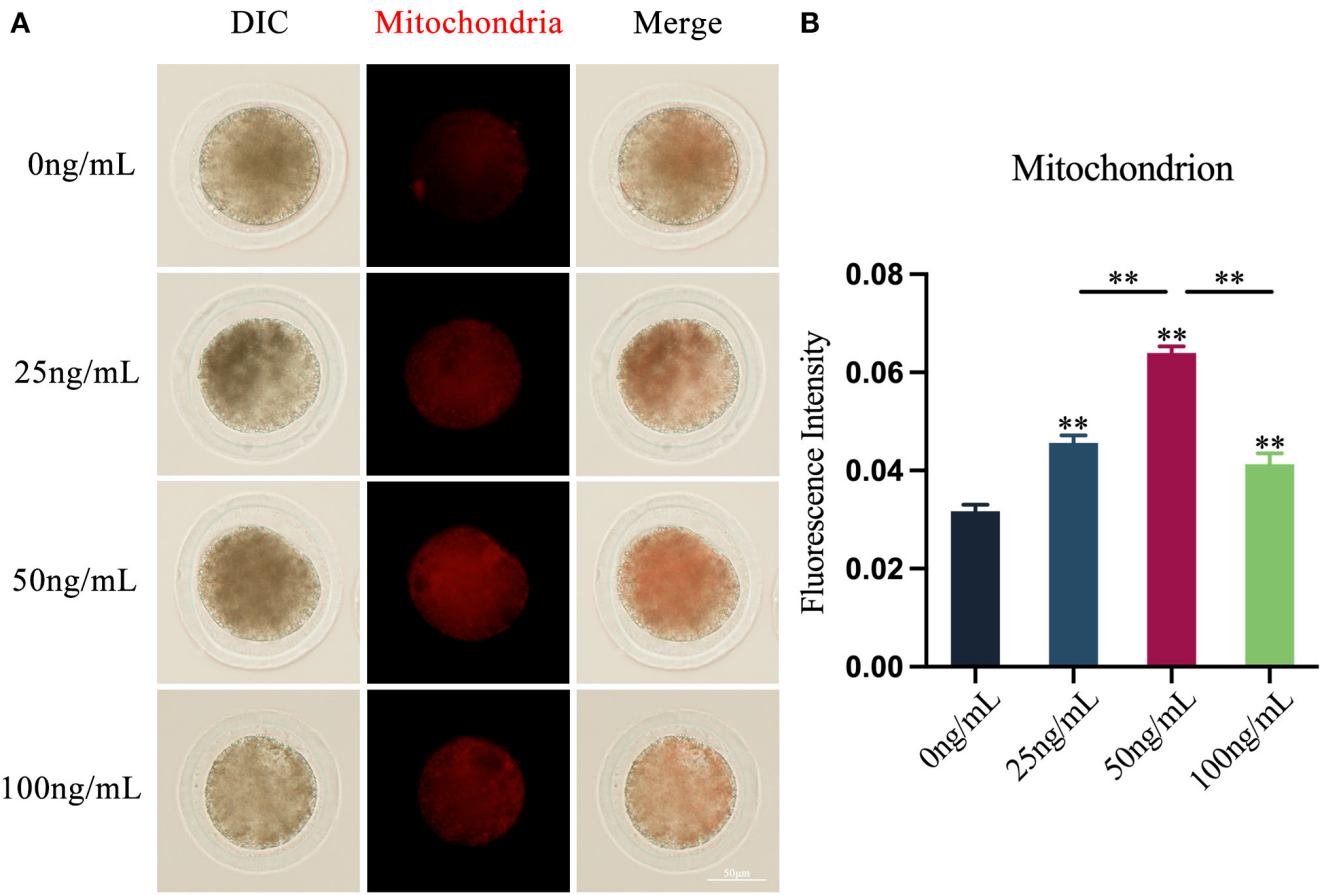
Effects of different concentrations of LIF on the distribution and expression of mitochondria in yak oocytes. **(A)** Effects of LIF on mitochondrial distribution in oocytes. Bar = 50 μm. **(B)** Analysis of the intensity of mitochondrial fluorescence in oocytes in each experimental group. Data results are presented as Mean ± SEM. **On the histogram indicates a significant difference (**p < 0.01).

By examining the distribution of mitochondria in oocytes, the results showed that LIF could improve the distribution of oocytes mitochondria (Figure 2A). The fluorescence intensity of oocytes mitochondria in the LIF-treated group was significantly higher than that in the control group (*p* < 0.01, Figure 2B), and the fluorescence intensity of oocytes mitochondria in the 50 ng/mL LIF-treated group was also significantly higher than that in the 25 ng/mL LIF-treated group and the 100 ng/mL LIF-treated group *(p* < 0.01, Figure 2B).

### Effects of LIF on ROS levels in yak oocytes

**Figure 3:**
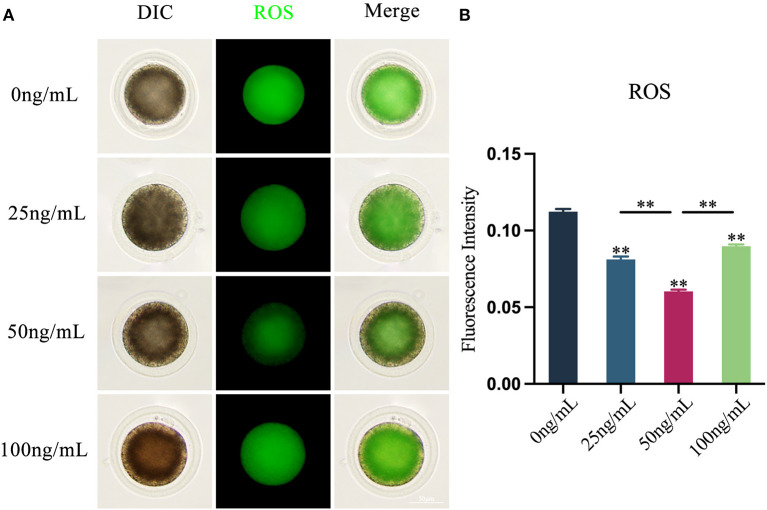
Effects of varying doses of LIF on ROS in yak oocytes. **(A)** Effect of LIF on ROS in oocytes. Bar = 50 μm. **(B)** Examination of the intensity of ROS fluorescence in oocytes in each experimental group. Data results are presented as Mean ± SEM. **On the histogram shows a significant difference (**p < 0.01).

The results revealed that adding varying amounts of LIF to the COCs maturation medium could diminish the production of ROS in oocytes (Figure 3A). The production of ROS in oocytes in the LIF-treated group was significantly lower than that in the control group (*p* < 0.01), and which in the 50 ng/mL LIF-treated group was also significantly lower than that in the 25 ng/mL LIF-treated group and the 100 ng/mL LIF-treated group (*p* < 0.01, Figure 3B).

### Effects of LIF on actin levels of yak oocytes

**Figure 4:**
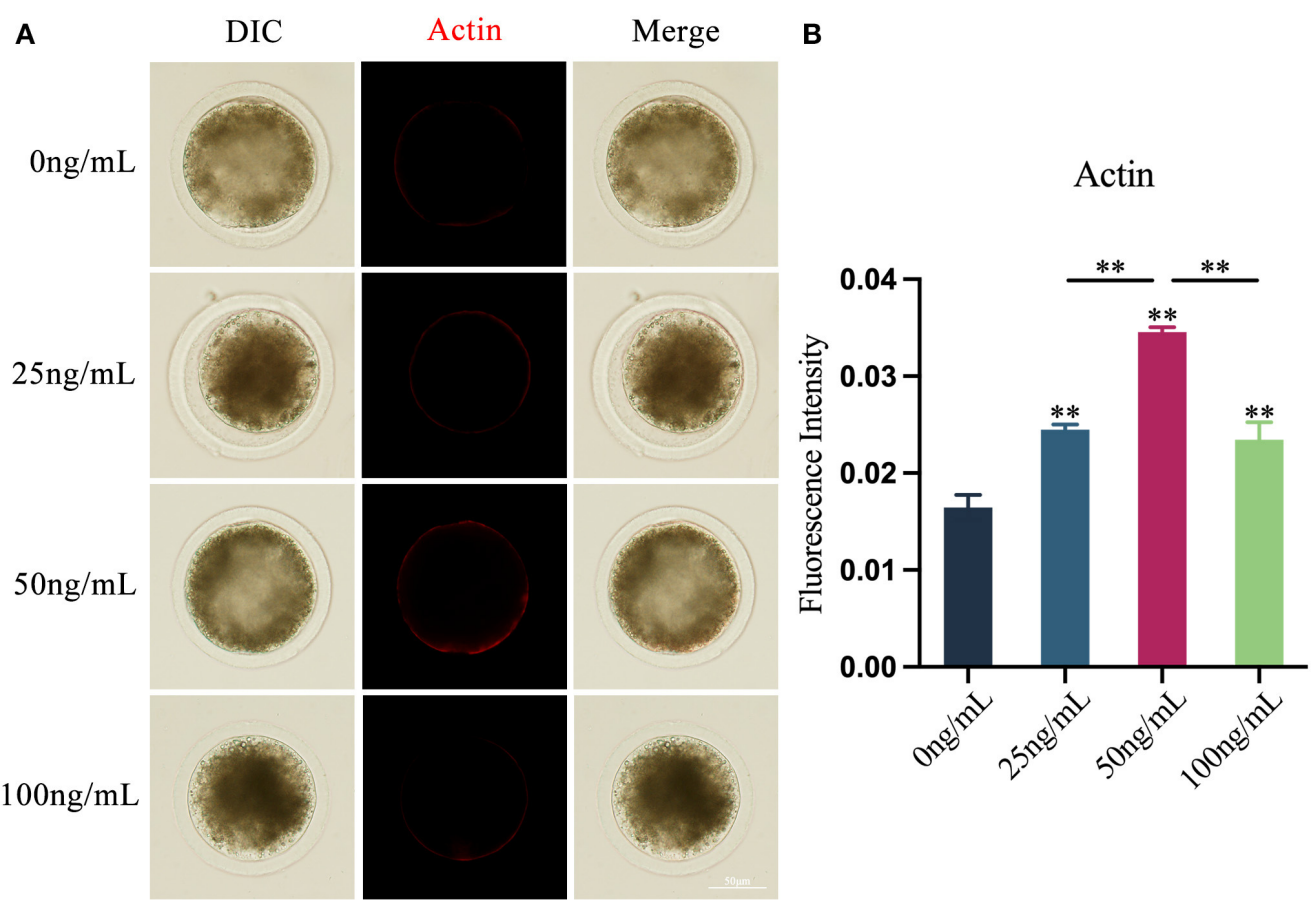
Effects of various doses of LIF on oocytes actin in yak oocytes. **(A)** Effects of LIF on oocytes actin integrity. Bar = 50 μm. **(B)** Analysis of oocytes actin fluorescence intensity in each experimental group. Data findings are reported as Mean ± SEM. **On the histogram shows a significant difference (**p < 0.01).

Using the Phalloidin-iFluor^™^ 594 Conjugate Kit, the results showed that LIF could improve oocytes actin integrity after adding various amounts of LIF to the COCs mature culture media (Figure 4A). The expression of actin in oocytes in the LIF-treated group was significantly higher than that in the control group (*p* < 0.01), and the expression of actin in oocytes in the 50 ng/mL LIF-treated group was significantly higher than that in the 25 ng/mL LIF treatment group and 100 ng/mL LIF-treated group (*p* < 0.01, Figure 4B).

### Effects of LIF on early apoptosis of yak oocytes

**Figure 5:**
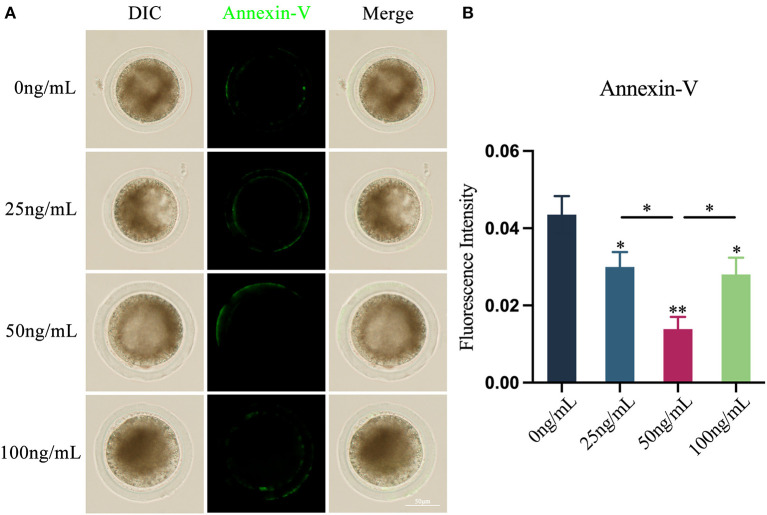
Effects of different concentrations of LIF on yak oocytes early apoptosis. **(A)** Effects of LIF on oocyte membrane apoptosis. Bar = 50 μm. **(B)** Analysis of the oocyte membrane's apoptosis fluorescence intensity in each experimental group. Data results are presented as Mean ± SEM. * or ** on the bar graph indicates a significant difference (*p < 0.05 and **p < 0.01).

Using Annexin V-FITC apoptosis detection kit, the presence of fluorescent signals on the cell membrane of oocytes is considered to that the early apoptosis occurs in oocytes (Figure 5A). The findings demonstrated that the addition of various quantities of LIF may lower the apoptosis level of oocytes (Figure 5B). The apoptosis levels of the oocytes in the 25 ng/mL LIF-treated group and the 100 ng/mL LIF-treated group were significantly lower than those in the control group (*p* < 0.05, Figure 5B). The apoptosis level of oocytes in the 50 ng/mL LIF treatment group was significantly lower than that in the control group (*p* < 0.01, Figure 5B), and also significantly lower than that in the 25 ng/mL LIF-treated and the 100 ng/mL LIF-treated groups (*p* < 0.05, Figure 5B).

### Effects of LIF on the binding ability of yak oocytes and sperm

**Figure 6:**
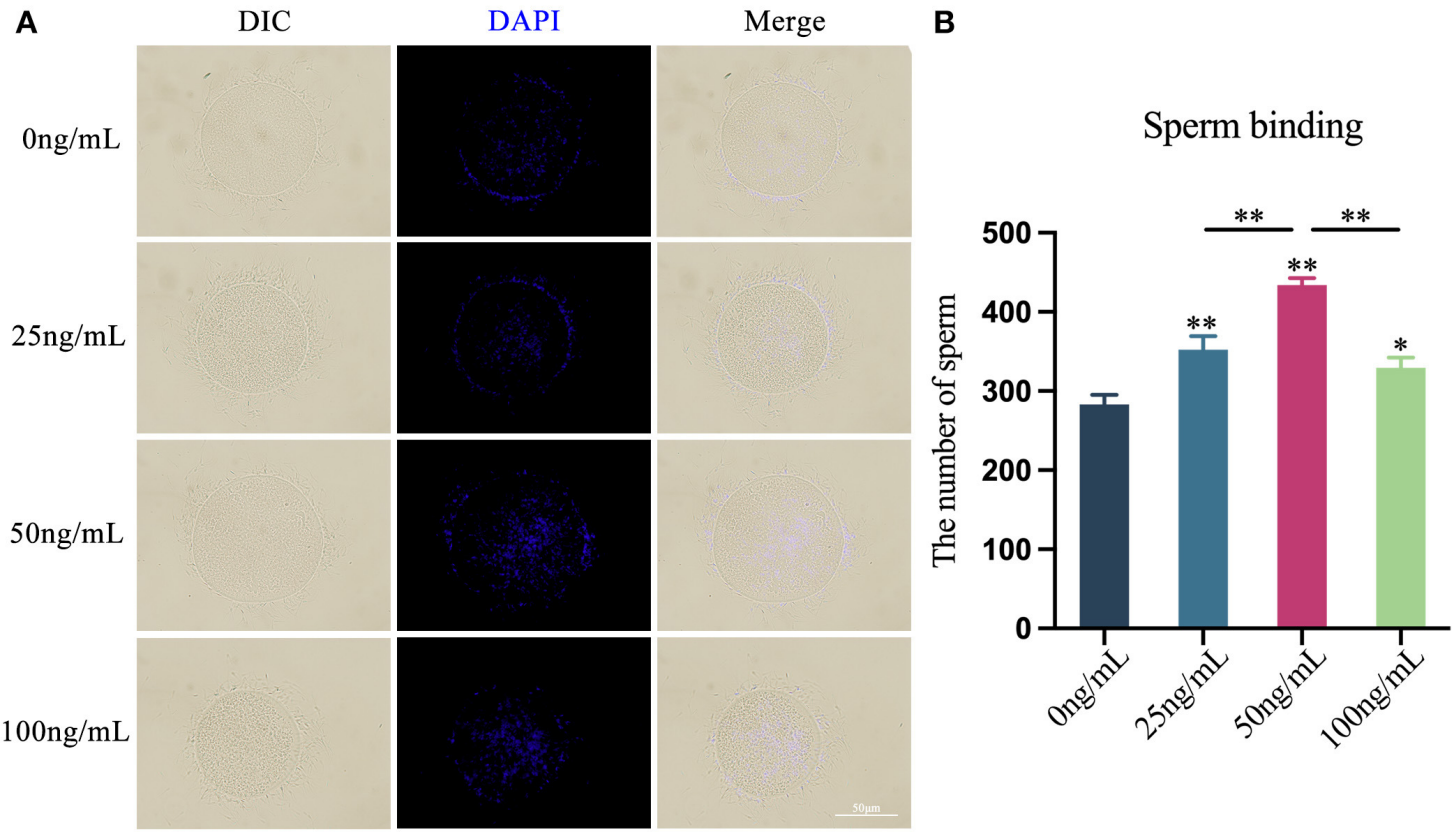
Effects of different concentrations of LIF on the binding capacity of yak oocytes and sperm. **(A)** Effects of LIF on oocytes and sperm binding capacity. Bar = 50 μm. **(B)** Analysis of the number of oocytes-bound sperm in each experimental group. Data results are presented as Mean ± SEM. * or ** on the bar graph indicates significant difference (*p < 0.05 and **p < 0.01).

Through the *in vitro* COCs-sperm binding experiment, after adding various doses of LIF to the COCs mature culture medium, the binding capacity of oocytes and sperm was enhanced (Figure 6A). The number of sperm bound to oocytes in the 25 ng/mL LIF-treated group was significantly higher than that in the control group (*p* < 0.01, Figure 6B), and the number of sperm bound to oocytes in the 100 ng/mL LIF-treated group was also significantly higher than that in the control group (*p* < 0.05, Figure 6B). The number of spermatozoa binding to oocytes in the 50 ng/mL LIF-treated group was significantly higher than that in the control, 25 ng/mL LIF-treated and 100 ng/mL LIF-treated groups (*p* < 0.01, Figure 6B).

### Effects of LIF addition during IVM on the apoptosis level and the total cell number of subsequent parthenogenetically activated blastocysts

**Figure 7:**
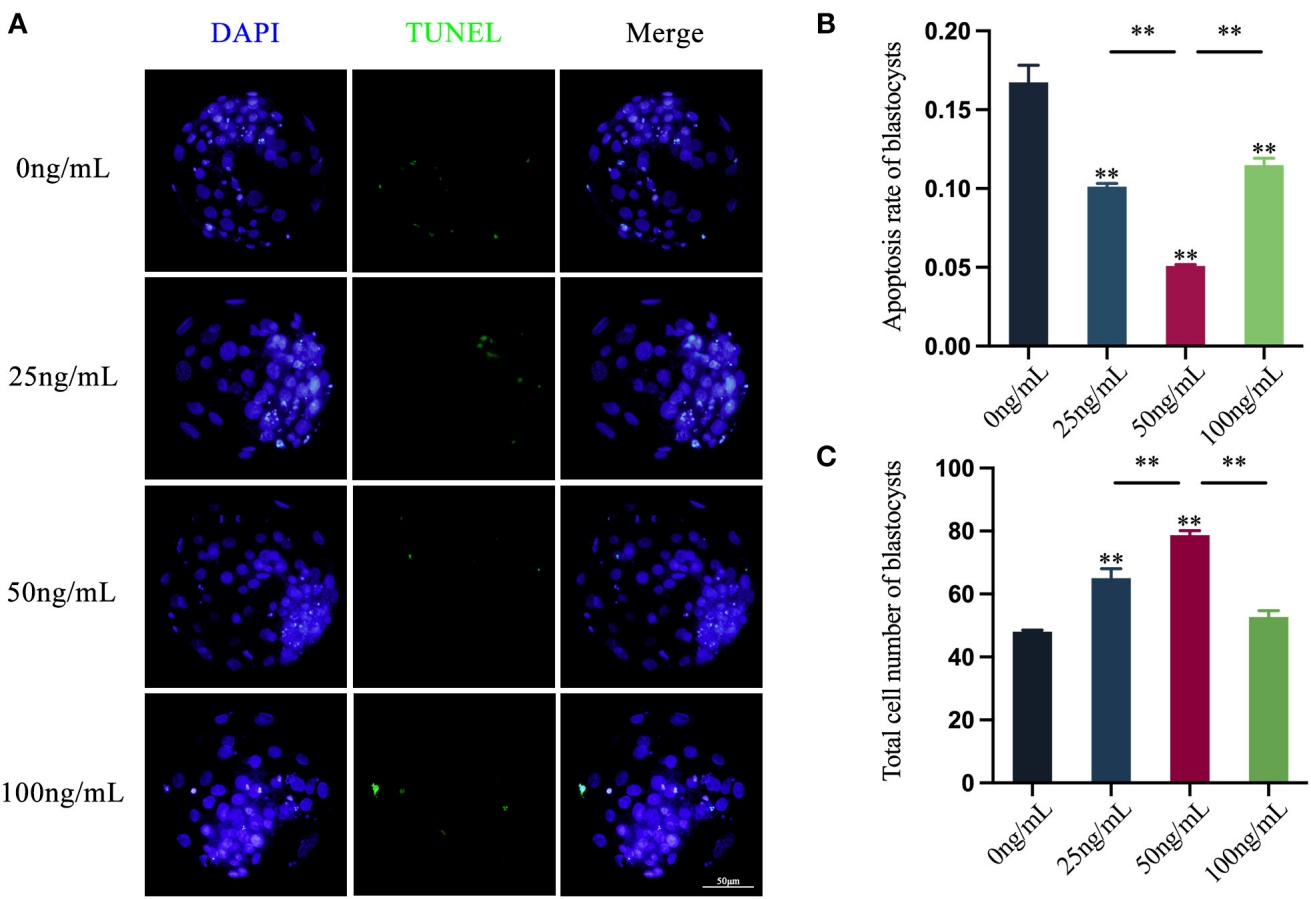
Effects of LIF addition during IVM on the level of apoptosis and total cell number in parthenogenetically activated blastocysts. **(A)** Effects of LIF on blastocysts. Bar = 50 μm. **(B)** Analysis of blastocysts apoptosis rate after parthenogenetic activation of oocytes in each experimental group. **(C)** Statistics of the total number of blastocysts after parthenogenetic activation of oocytes in each experimental group. Data results are expressed as Mean ± SEM. **On the histogram indicates significant difference (**p < 0.01).

Using the *In Situ* Cell Death Detection Kit, the presence of fluorescent signals on the nucleus of the blastocyst is considered to that apoptosis occurs in the blastocyst.. Oocytes treated with different concentrations of LIF could reduce the apoptosis level of subsequent blastocysts (Figure 7A). The blastocysts apoptosis rate of each experimental group was counted and the results showed that the blastocyst apoptotic rates in the 25 ng/mL LIF-treated group and the 100 ng/mL LIF-treated group were significantly lower than those in the control group (*p* < 0.01, Figure 7B). The apoptotic rates of blastocysts in the 50 ng/mL LIF-treated group were significantly lower than those in the control, 25 ng/mL LIF-treated and 100 ng/mL LIF-treated groups (*p* < 0.01, Figure 7B).

The total cell number of blastocysts in each experimental group was counted and the results revealed that the total cell number of blastocysts in the 25 ng/mL LIF-treated and the 50 ng/mL LIF-treated groups were significantly higher than that in the control group (*p* < 0.01, Figure 7C). However, the total cell number of blastocysts in the 100 ng/mL LIF treatment group was not significantly different from that in the control group (*p* > 0.05, Figure 7C). The total cell number of blastocysts in the 50 ng/mL LIF-treated group was extremely higher than that in the 25 ng/mL LIF-treated (*p* < 0.01) and the 100 ng/mL LIF-treated groups (*p* < 0.01, Figure 7C).

### Effects of LIF on the expression of genes mRNA in yak oocytes

**Figure 8:**
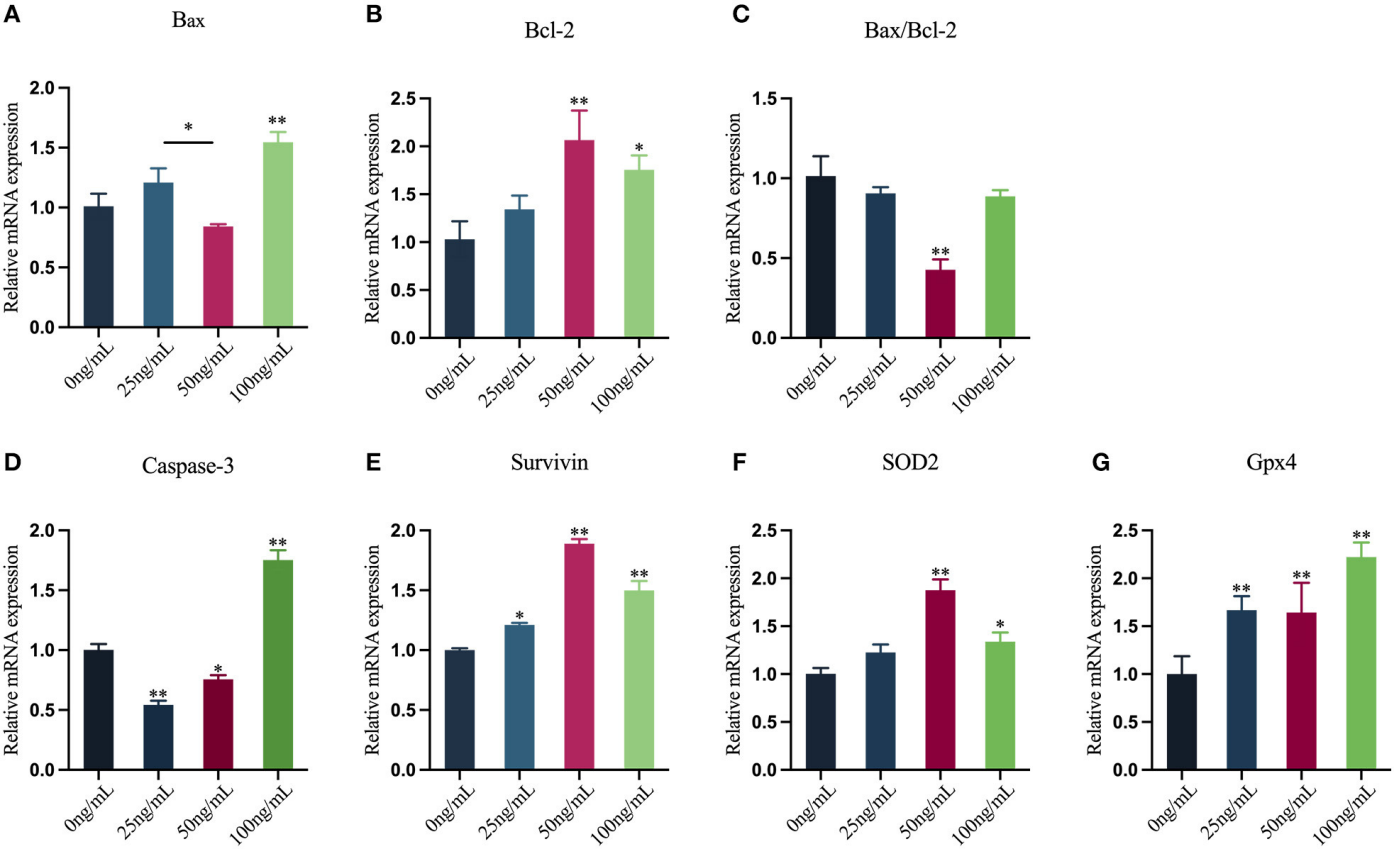
Effects of varying doses of LIF on the mRNA expression levels of yak oocyte-related genes. **(A–E)** Apoptosis-related gene expression [**(A)** *Bax*, **(B)** *Bcl-2*, **(C)** *Bax/Bcl-2*, **(D)** *Caspase-3*, **(E)** *Survivin*]. **(F,G)** Antioxidant-related gene expression [**(F)** *SOD2*, **(G)** *Gpx4*]. Data results are reported as Mean ± SEM. * or ** on the bar graph shows that the findings are substantially different (*p < 0.05 and **p < 0.01).

In this study, qRT-PCR was used to assess the mRNA expression levels of oocytes apoptosis-related genes and antioxidant-related genes (Figures 8A–G). The results showed that the mRNA expression of *Bax* in oocytes in the 25 ng/mL LIF-treated and the 50 ng/mL LIF-treated groups were not significantly different from that in the control group (*p* > 0.05), but the mRNA expression of *Bax* in oocytes in the 100 ng/mL LIF-treated group was significantly higher than that in the control group (*p* < 0.01). The mRNA expression of *Bax* in oocytes in the 50 ng/mL LIF-treated group was significantly lower than that in the 25 ng/mL LIF-treated group (*p* < 0.05). The expression of *Bcl-2* mRNA in oocytes in the 50 ng/mL LIF-treated group was substantially greater than that in the control group (*p* < 0.01), while the expression of *Bcl-2* mRNA in oocytes in the 100 ng/mL LIF-treated group was significantly higher than that in the control group (*p* < 0.05), whereas the expression of *Bcl-2* mRNA in oocytes in the 25 ng/mL LIF treatment group was not statistically different from that in the control group (*p* > 0.05). The *Bax/Bcl-2* mRNA expression ratio of oocytes in the 50 ng/mL LIF-treated group was significantly lower than that in the control group (*p* < 0.01), and the *Bax/Bcl-2* mRNA expression ratio in oocytes in the 25 ng/mL LIF-treated and 100 ng/mL LIF-treated groups was not significantly different from the control group (*p* > 0.05). The expression of *Caspase-3* mRNA in oocytes of the 25 ng/mL LIF-treated and the 50 ng/mL LIF-treated groups was significantly lower than that of the control group (*p* < 0.01, *p* < 0.05), but the expression of *Caspase-3* mRNA of oocytes in the 100 ng/mL LIF-treated group was significantly higher than that of the control group (*p* < 0.01). The mRNA expression of *Survivin* in oocytes in the 50 ng/mL LIF-treated and the 100 ng/mL LIF-treated groups was significantly higher than that in the control group (*p* < 0.01), while the mRNA expression of *Survivin* in oocytes in the 25 ng/mL LIF-treated group was significantly higher than that in the control group (*p* < 0.05). The expression of *SOD2* mRNA in oocytes in the 50 ng/mL LIF-treated and the 100 ng/mL LIF-treated groups was significantly higher than that in the control group (*p* < 0.01, *p* < 0.05), but the expression of *SOD2* mRNA in oocytes in the 25 ng/mL LIF-treated group was not significantly different from the control group (*p* > 0.05). The expression of *Gpx4* mRNA in oocytes of the 25 ng/mL LIF-treated group, 50 ng/mL LIF-treated group, and 100 ng/mL LIF-treated group was significantly higher than that of the control group (*p* < 0.01).

## Discussion

Previous studies have demonstrated that LIF may enhance the developing competence of oocytes in mammals such as mice, pigs, cattle and sheep (24, 26, 33, 34). Therefore, we hypothesized that LIF could improve the development and maturation of yak oocytes. To prove this hypothesis, we examined the rate of the first polar body of oocytes and the ability of sperm-egg binding, which are important indicators of oocyte quality and viability and the key steps of fertilization (35). The results showed that LIF could improve the quality of yak oocytes *in vitro* maturation. Meanwhile, the effect of 50 ng/mL LIF on oocytes was significantly higher than that of 25 ng/mL LIF and 100 ng/mL LIF. Therefore, we concluded that yak oocytes have different sensitivity to different concentrations of LIF. We then examined the quality of subsequent blastocysts, another indicator of oocytes quality. The results showed that the blastocysts after parthenogenetic activation of oocytes treated with 50 ng/mL LIF had higher total cell number and lower apoptosis rate than those treated with 25 ng/mL and 100 ng/mL LIF, which proved the previous conclusion.

To explore the potential regulatory mechanism, we examined the effects of LIF on oocytes early apoptosis, genes, mitochondria, ROS and actin. The findings of this experiment revealed that LIF could lower the apoptosis level of oocytes membranes to promote the maturation and development of oocytes. In addition, the gene expression data showed that LIF might regulate the mRNA expression of apoptosis-related genes (*Bax, Bcl-2, Survivin, Caspase-3*) to reduce the early apoptosis level of oocytes. Moreover, LIF could raise the mRNA expression levels of oocyte antioxidant genes (*SOD2, Gpx4*) to resist oxidative stress damage to oocytes. Therefore, LIF could regulate genes expression to improve oocytes maturation and development *in vitro*.

The findings of this experiment demonstrated that LIF could modify the distribution of mitochondria in yak oocytes and increase their amount. Oocytes are the biggest cells in mammals. Mitochondria are key organelles producing energy inside the cell (8), occupy a large fraction of the cytoplasm of the oocyte (36) and affect the developmental potential of the oocytes (37). Therefore, impaired mitochondrial activity lowers oocytes developmental ability (38). Mitochondrial supplementation in human, mouse, porcine, and bovine oocytes is found to increase egg quality and *in vitro* fertilization success (39–41), and restores preimplantation embryonic developmental competence (38). The quantity of mitochondrial storage directly impacts the amount of ATP. Numerous events such as transcription, translation, and spindle assembly during oocytes maturation, fertilization, and preimplantation embryos development require considerable quantities of ATP (36, 42). Decreased ATP generation may impair processes such as cell cycle regulation, spindle formation, fertilization, and preimplantation embryos development (36). Furthermore, mitochondria may mediate the endogenous apoptotic pathway in mammalian oocytes (43). Mitochondria play a crucial role in apoptosis by modulating signaling molecules such as calcium Ca^2+^, cyclic adenosine monophosphate (cAMP), and ROS (5, 12). Calcium and Bcl-2 family proteins are involved in the process of apoptosis (44). When calpain is activated, intracellular Bcl-2 protein expression drops, which in turn stimulates mitochondrial apoptosis (13). Therefore, we concluded that LIF can improve the quality and developmental ability of oocytes by regulating the function of mitochondria, and then affect the quality of subsequent blastocysts *in vitro*.

The findings of this experiment demonstrated that LIF could lower the production of ROS in yak oocytes and maintain a reasonably low level of ROS. ROS is a product of oocyte metabolism, and excess ROS may contribute to oxidative stress (5). ROS are also crucial signal transduction molecules that play a critical role in regulating cellular metabolic processes by oxidizing protein cysteine residues to promote proliferation, differentiation, and migration (36). Oxidative stress may oxidize RNA, DNA, and proteins, altering the structure of cell membranes and consequently affecting oocyte development (45). In addition, ROS are very directly associated with the activation of mitochondrial pathways (44). Most of the intracellular ROS is created owing to leakage of the respiratory electron transport chain (46). ROS damage mitochondrial DNA, which in turn impairs the transcription of mtRNA proteins involved in the electron transport chain, leading to respiratory chain dysfunction and further production of ROS, resulting in loss of mitochondrial membrane potential and impairment of ATP synthesis (44), which in turn affects oocyte and embryonic developmental capacity (47, 48). Therefore, we concluded that LIF can reduce the level of ROS and resist the damage of oxidative stress to mitochondria, RNA, DNA and proteins in oocytes, thereby promoting the maturation and development of oocytes *in vitro*.

The findings of this experiment revealed that LIF could enhance the integrity of actin and raise the amount of actin. During mammalian oocytes maturation, it is required for the oocytes to undergo two asymmetric divisions (49). This asymmetric division protects the cytoplasm, comprising essential resources such as mRNA, proteins, and mitochondria, to promote early embryonic development (21). Actin plays a crucial function in this asymmetric division. Firstly, actin plays a vital function in spindle movement and cortical polarization during oocyte meiosis (50). Secondly, in a mouse model, dynamic actin filaments around the meiotic spindle push mitochondria, thus producing a counterforce to spindle migration in oocytes (51). Therefore, we concluded that LIF can affect actin to ensure the normal meiosis of oocytes, thereby promoting the development of oocytes *in vitro*.

In summary, the results of this study showed that adding LIF (50 ng/mL) treatment during the *in vitro* maturation of yak oocytes could enhance mitochondrial and actin levels in oocytes, reduce ROS and apoptosis levels and regulate the mRNA levels of related genes, thereby promoting the maturation rate of oocytes and their ability to combine with sperm under *in vitro* culture conditions. In addition, LIF-treated oocytes could lower the apoptosis rate of subsequent blastocysts and increase the total cell number of blastocysts.

## Conclusion

This study explored the effect of LIF on the *in vitro* maturation of yak oocytes. LIF decreased oocyte apoptosis by modulating mitochondria, oxidative stress and actin levels in yak oocytes, consequently enhancing the quality of yak oocytes, promoting the *in vitro* maturation and development of yak oocytes and the quality of subsequent blastocysts. The result of this study provided some supportive insights into the role and function of LIF *in vitro* yak oocytes maturation, as well as provided fundamental knowledge for assisted reproductive technologies in the yak.

## Data availability statement

The original contributions presented in the study are included in the article/supplementary material, further inquiries can be directed to the corresponding author/s.

## Ethics statement

The animal study was reviewed and approved by the Experimental Animal Ethics Committee of Gansu Agricultural University (Ethic approval file No. GSAU-Eth-VMC-2022-23).

## Author contributions

TZhao, YP, YC, and SY came up with and designed the study. JW and TZhan collected samples. TZhao, QL, and TD performed the experiments. TZhao, YW, LZ, and XH performed the statistical analysis. TZhao wrote the manuscript. YP, RN, AB, and SY revised the manuscript. All authors contributed to the article and approved the submitted version.

## Funding

This research was funded by the National Natural Science Foundation of China (Grant Nos. 31972760 and 32160850), the Fund for Distinguished Young Scholars of Gansu Province (Grant No. 20JR10RA561), the Science and Technology Major Project of Gansu Province (Grant No. 21ZD10NA001), the Seed Industry Research Project of Gansu Province (Grant No. GZGG-2021-1), and the Key Talent Project of Gansu Province (2022-0623-RCC-0307).

## Conflict of interest

The authors declare that the research was conducted in the absence of any commercial or financial relationships that could be construed as a potential conflict of interest.

## Publisher's note

All claims expressed in this article are solely those of the authors and do not necessarily represent those of their affiliated organizations, or those of the publisher, the editors and the reviewers. Any product that may be evaluated in this article, or claim that may be made by its manufacturer, is not guaranteed or endorsed by the publisher.
